# Cases of Progression Locomotor Ataxia

**Published:** 1867-03

**Authors:** James Brown Burnet

**Affiliations:** House Physician, Bellevue Hospital, New York


*Cases of Progrsssive Locomotor Ataxia.* By James Brown Burnet, M. D., House Physician, Bellevue Hospital, New York.

Case 1. Robert Gardner, 35 years of age, was born in Ireland, of healthy parents. He has been in this country for eighteen years, and has been a butler by occupation. He always has enjoyed good health up to about twelve years ago, when in the winter season he was taken with a violent pain in the small of his back, which pain extended around, principally on the left side. These pains would last two or three days, then pass away, and he would feel as well as ever. During the winter seasons these attacks would come on three or four times. The physicians pronounced his disease to be lumbago, and treated him with Rochelle salts. He would have at the same time quite a severe pain in the præcordial region, which would pass off with a pain in the back. During the summer season he enjoyed good health, never being troubled with these attacks.

About fifteen years ago he had intermittent fever, which lasted nearly six months, and from which he entirely recovered. A year ago last July, whenever he went out into the street, he would be seized with dizziness, which would pass off when he came into the house. He also was troubled with headaches, which would come on in the morning and last until about 12 o'clock. These would probably take place two or three times a month, and were very violent while he had them. During these attacks he would have an eruption on his head, which would last as long as the pain, and then in a day or two scale off. About the middle of October, his feet would at times become numb and cold, especially at night, and very violent pains would commence at the knees, go down to the feet, and then ascend to the head, these pains sometimes lasting the whole night. These would continue for three or four nights, and then go off for a week or so. Any change in the weather would seem to bring them on.

On the second of January he entered St. Luke's Hospital, and was there treated with strychnia. This continued until March, when he became quite weak, so that he was unable to walk. His medicine was then changed to Rochelle salts, and afterward back again to strychnia, but still his legs continued weak, and the pain was as severe as ever.

About fifteen years ago he had gonorrhœa, which lasted nine months. He also had a small sore on his penis, and a bubo, but no breaking out on his body at this time. About six years ago he was troubled with a stricture, and again while at St. Luke's Hospital, so that it was necessary to draw his water from him. This lasted for about one month. About four years after he had the sore on his penis, he had a slight breaking out on both arms, and afterward on his head. On the 11th of June he left the hospital on crutches, feeling no better. He remained with a friend until the 21st of July, when he was admitted to this hospital, not being any stronger in his limbs. He at this time complained of severe pain in the lower limbs, and partial paralysis. There was permanent contraction of both pupils. They acted very sluggishly when exposed to a strong light. Slight tingling sensations in the arms; sensation not impaired, but it was slightly impaired in the lower extremities. The left was the worst leg. At the present time he has a numb feeling always before passing his water, and his penis often feels hot, which sensation partially passes off after he urinates. Whenever he lies on his belly, he has a dull heavy pain in the back, over the region of his kidneys. Since he has been in the haspital he has been gradually improving. There has been a decided improvement in his walk, as he can now go a short distance without the assistance of a cane. He walks cautiously, however, and watches each foot as it is raised from the floor. He falls backward if he attempts to stand still with closed eyelids. Whenever he stands upright he experiences pain in the lower part of his back. He has no pain in his back, except he lies on his face or attempts to stand straight. His urine has been examined, and found to be normal.

The diagnosis was made of *progressive locomotor ataxia*, and the treatment consisted in the administration of potassii iodidi, ten grains three times a day, with fifteen drops of syr. ferri iodidi. Bowels have been kept regular with pills of aloes, nux vomica, and belladonna, in small doses. His back and limbs have been vigorously rubbed with lin. sap. camph. He is gradually improving.

Oct. 29th. He appears to remain about in *statu quo*. If anything, the pains in the limbs are rather worse.

Nov. 1st. He complains of lascivious dreams and seminal emissions, which appear to weaken him considerably. He has them regularly as often as once a week, and at times much oftener. He has had these for a number of years. He has formerly indulged himself in venereal excesses to a considerable extent, but never practiced masturbation. He has been ordered ten grains of potassi bromidi each night before retiring.

Nov. 2d. He could not sleep any last night on account of excruciating pains darting along his tibia. There are no nodes, and no symptoms of syphilitic periostitis.

Affairs thus went on until the early part of winter, when he was discharged, with very little change in his general condition.

Case 2.—Anna Farly, 32 years of age, single, and a native of Ireland, was admitted to ward 14 of Bellevue Hospital on September 29th, 1866. She gave the following history of herself. Her father was healthy, and died at the advanced age of 87; her mother still lives, is nearly 80, and suffers with asthma. Anna has resided in this country 16 years, and her occupation is to cook, wash, and iron. She always enjoyed good health until she had been in this country about one month, when she was laid up with typhus fever in Cincinnati for about eight weeks. In the winter of 1852, she went to New Orleans, where she took the yellow fever, from which she did not fully convalesce for six months. In the summer of 1855, she went to Mobile, and was on a plantation, where she was taken with chills and fever, which lasted two weeks; then they would pass of for a few weeks, and then come on again, and so on for a whole year. During these attacks she had violent attacks of epistaxis. She rarely had such attacks before, and never but once since. After this she had good health until about three years ago, when at a party one evening, she was seized with a violent shivering throughout her whole system, which lasted only a short time however; but since that time she has been extremely nervous. At about the same period she would have attacks of dizziness, and quite a severe pain appeared in the posterior part of the head, sometimes being dull and heavy. This continued a few months, when she felt a pricking sensation coming on in the head, and gradually passing down to the feet, sometimes severe, and then again quite slight. In about six months after the first attack she was afflicted with double vision, which lasted nearly four months without ceasing. At the end ot this time she had a very violent pain in the small of her back, which extended all around the body, and also all over the thorax, and as soon as this took place the double vision ceased. These sensations and numb feelings have remained with her up to the present time. About a year ago, the dizziness became so marked that it was almost impossible for her to walk at all, and she became very weak. For the past year, also, dull pains have been in her feet, which feel quite cold and occasionally numb. She is quite nervous now, and sometimes is troubled with a twitching of the muscles of the face. For the last eight years she has noticed that, when she caught cold and coughed, or vomited from any cause, she would throw up more or less blood. This at first would be clotted and dark, but afterward of a lighter red color, mixed with the sputa. She has a slight cough, especially in the morning, and sometimes a slight expectoration. Her appetite is capricious. For two years she has been troubled with a leucorrhœal discharge. During the past year and a half, she has also been troubled off and on with a difficulty in urinating, sometimes going 24 hours without being able to pass her urine. This was always relieved by nitre. She never was obliged to have her urine drawn by a catheter. She generally has been costive in her bowels. In three or four days after admission to the hospital, she was taken with a severe diarrhœa, for which she was treated with the following prescription:

| | | | |
|---|---|---|---|
| ℞ | Bismuthi subnitrat., | ʒj | |
| | Pulv. opii., | gr. x. | |
| | Acidi tannici, | ʒss. | M. |

Et div. in chart, no x.

S. One three times a day.

And from which diarrhœa she rapidly recovered. Generally in the afternoon her feet and legs begin to swell. This has been so for nearly two years. Pupils act well to light. Sen sation is not impaired. She walks with an unsteady gait.

If she stands still and closes her eyes, she feels as though she was swaying from side to side. She can grasp the hand with a firm grip. There seems to be great weakness in her back. She used to have great palpitation of the heart, but has not felt this for the past year. Her urine has been examined and found to be normal. Evidences of incipient tuberculosis. The diagnosis was made of *progressive locomotor ataxia*, and the treatment commenced, was 10 grains of the iodide of potassium, three times a day, in syrup of sarsaparilla. She is also taking tr. ferri muriat. She thinks there is no improvement. She has had sore-throat, and her hair has fallen out, but she has never had any eruption on the body save prickly heat. She denies all syphilitic history.

Oct. 29th. No improvement. She has stopped taking potassi iodidi, and is now taking three times a day, one sixth of a grain of the nitrate of silver.

Nov. 1st. Feels rather worse. The pains in the head are increased. Locomotor powers are no better.

Dec. 1st. Discharged no better.—*Med. & Surg. Reporter.*

